# Supplementary figures and images for: Investigating barriers & facilitators for the successful implementation of the BP@home initiative in London: Primary care perspectives
Source: PLoS One. 2024 Feb 29;19(2):e0298898. doi: 10.1371/journal.pone.0298898 (PMC10903909; doi:10.1371/journal.pone.0298898)

**S1 Fig. UCLP High Blood Pressure Stratification and Management Framework**


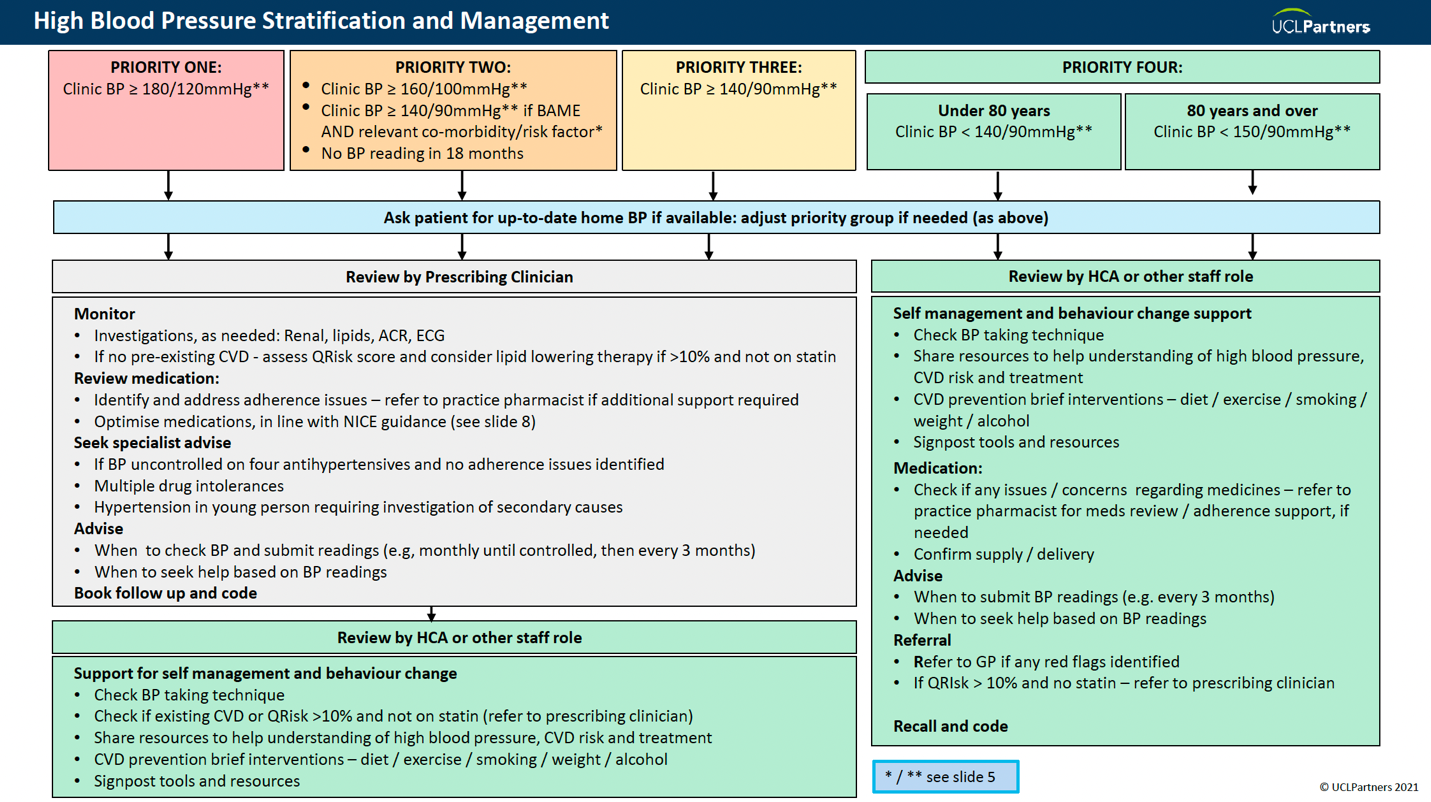

Supplement: S1 Fig — (DOCX) [file pone.0298898.s001.docx]

**S2 Fig: BPM allocation recommended pathway**


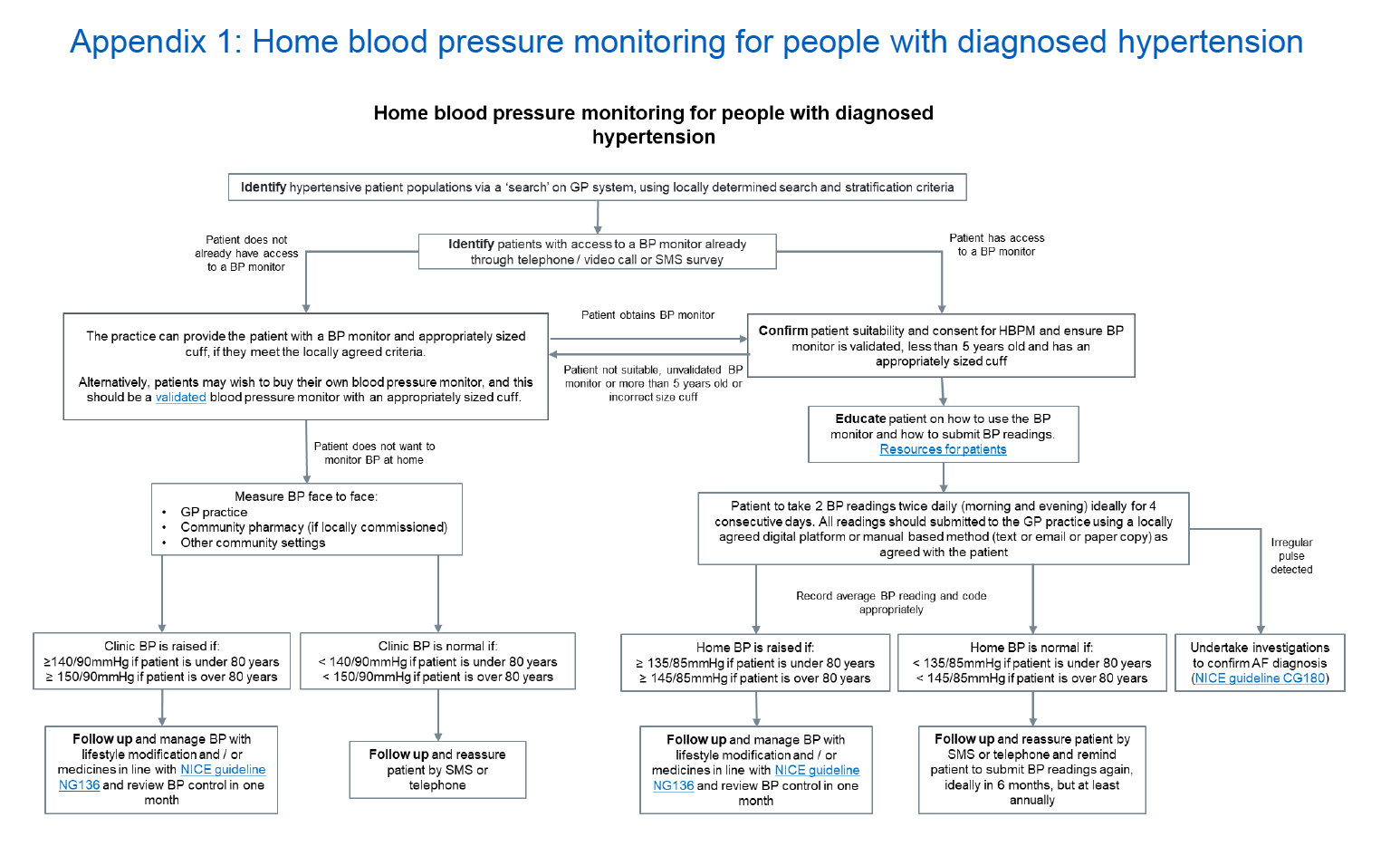

Supplement: S2 Fig — (DOCX) [file pone.0298898.s002.docx]
