# Supplementary material for: Investigating barriers & facilitators for the successful implementation of the BP@home initiative in London: Primary care perspectives
Source: PLoS One. 2024 Feb 29;19(2):e0298898. doi: 10.1371/journal.pone.0298898 (PMC10903909; doi:10.1371/journal.pone.0298898)
Supplement: S1 Table — (DOCX) [file pone.0298898.s003.docx]

**S1 Table: Interview Guide**

| **Respondents** | **Questions** |
| --- | --- |
| **Commissioners, decision makers / PCN** | - Was there sufficient communication & awareness about BP@Home initiative? - Were there funding & evaluation plans suggested? - Were the roles of the different actors involved discussed / clarified (NHSE, Evaluation Cell, ARC, ICSs)? Was there central guidance issued? - How did you plan and manage the receipt & storage of BP monitors? Could you tell us about the logistics? - How did you envision IT/data infrastructure at ICS level will be leveraged to support quant evaluation? - Which IT platform for BP@Home data collection from patients did you recommend using and why (e.g. AccuRx, Huma)? How did you come to this decision? - How did you identify participating PCNs, practices and GPs in your network? - BP@Home focuses on patients with known hypertension who are shielding and remain poorly controlled with a last recorded blood pressure of systolic BP > 150mmHg and / or diastolic BP > 90mmHg. Did your region follow these NHSE criteria for allocation? - How did you approach inequalities aspect of this project? |
| **PM staff** | - How was information about the initiative received? Was there an opportunity for your team to co-create local solutions for implementation? - Did you use the NHSE criteria for allocation? How about UCLP? Operationally, how easy/difficult was it to implement these? - If all eligible patients cannot receive a BP monitor, whom do you prioritise & how? - How do you address issues of inequalities (accessibility, digital literacy) - How would you describe the culture of decision making in the real world setting for this programme given other emergent prioritise (e.g. COVID vaccinations)? - What would you propose to promote greater awareness and equitable provision of BPM? What recommendations could you make to make the process more person-centered? - How did your PCN/practice contact patients? Email, SMS, calls, F2F? Was this in phases? - What do you think are the main challenges to successful implementation of such initiatives? What would you change for future programs? - How would your practice/PCN audit the data to determine if participating in BP@H or other relevant initiatives is having an impact? |
| **Patient-facing HCPs** | - How & Who typically engaged with patients to deliver the BP@Home program? - How would you describe the onboarding process? To what extent was this person-centred? - Did you have access to any specific onboarding & educational material? Provenance? Any additional in-house material or external resources? How helpful & adequate are these? What would you change? - Have you had any training on how best to onboard/support patients enrolled in BP@H? What was the modality of training? How useful did you find it & what else would you recommend is included? - How empowered do you feel to deliver proactive care agenda, personalised care, person-centredness generally & for BP@H specifically - Do you have examples or case studies of patients who refused? Completed onboarding well but did not report readings, examples of very successful interaction post onboarding - How do you follow patient readings? If patients do not use the online platform, how do you access the readings? - Was there workflow support for managing patient readings (treatment & follow-up plans)? - Do you feel that your ability to support self-care could be improved, and if so, how? How did you follow up with non-respondents? - What were the main barriers & drivers in the process? What would you change? |
