# Supplementary material for: Investigating barriers & facilitators for the successful implementation of the BP@home initiative in London: Primary care perspectives
Source: PLoS One. 2024 Feb 29;19(2):e0298898. doi: 10.1371/journal.pone.0298898 (PMC10903909; doi:10.1371/journal.pone.0298898)
Supplement: S3 Table — (DOCX) [file pone.0298898.s005.docx]

**S3 Table: Thematic analysis of barriers & challenges identified by respondents (with supporting quotes)**

| **Categories** | **Themes** | **Key Supporting Quotes** |
| --- | --- | --- |
| **(1)**  **Project Management** | 1. **Shifting directions & lack of guidance** | - *It started with being a project about working with UCLP. It then sort of got honed down to a project about hypertension. So a lot of the time, actually the funded time was lost doing other things before somebody decided this is what they wanted us to do. And then when that funding ran out, I think each of the boroughs in our ICS have to try to keep on a fellow, but I think it's all being funded differently and everybody's got different amounts of money with a different label or a different hat on it. [PM]* - *You asked about the SOP and whether it was that amazing. No it wasn't. And we spent probably about four months in fortnightly meetings discussing and tweaking and getting a plan everybody was happy with before we launched. (…) So you see a lot of time and planning went into it and then once the SOP was finalised, it's just rolled the whole way through pretty well. So we did spend quite a lot of time drilling into the logistics of how specifically this is going to work [PM]* |
|  | 1. **Lack of resources (time, human, etc)** | - *Capacity within primary care is the main challenge [PM]* - *Getting dedicated time to do the work has definitely a barrier in many of the practices. [PM]* - *It requires a lot of admin and “old school chasing”. It is a full-time job to then chase the readings. It is an “ongoing chase of results” [GP]* - *(…) we needed to dedicate a member of staff, our trainee pharmacist, to really oversee it and push that aspect of care. Beyond that we got a team of pharmacist so it was quite easy then disseminating the work to them but the real difficulty was having someone really dedicated to oversee. [CP]* |
|  | 1. **Top-down approach & high staff turnover** | - *I feel that I've had very little ability to influence things at the ICS level. You know, we've had lots of frustrations [GP]* - *I think I kind of echo what what was said at least in terms of influence of the [local PCN] level, I you know, we received some communications every now and then that was that was it. [CP2]* - *with the delays and the BP machines and you know, no matter how many times we've kind of raised those concerns, nothing has really changed. [GP]* - *The difficulty is the turnover of staff that all practices have. So you know, in a training practice you've got new registrars every six months. They don't know about it. And are you gonna have a meeting every six months to say please record all this stuff here or do you just want them to get on with the managing people's blood pressure? So it's difficult to keep this stuff happening. [PM]* - *People are changing a lot in the ICS. So you know, we have had some influence with some people in NCL. And then they've moved on to different jobs and a lot of that work has been lost and the organisational memory got lost. [PM]* |
| **(2)**  **Logistics** | 1. **Storage & distribution of BPMs** | - *Delivery of 4,500 BPMs which created a bit of a panic. Pretty chaotic as a program. Distributed in a very unprofessional manner. [PM]* - *There was a significant error with the application for BP Monitors for [local ICS’. All monitors allocated in the third wave were redistributed to other areas leaving our ICS without any. As a result, we were tasked with procuring another cache of devices. This task was complicated and time consuming. Some primary care sites are still waiting for BPMs.[PM]* - *the hassles that we've had about getting monitors to practices and where they're going to store hundreds of monitors and you know that process has been a bit of a disaster and continues to be a bit of a disaster [PM]* - *the ICP procured the monitors and then said right, we're giving you a batch of monitors. So here come 3000 monitors. But then we had a very short window of time. Where we gonna put all these monitors? So trying to then coordinate between different practice sites that you're gonna have all this massive storage of of monitors. And actually there's now like 2 office rooms at the federation that are just chock full of monitors (…)Some monitors still in boxes because not all PCNS were ready at the same time. [PM]* - *Only one XL cuff per practice [PM]* |
|  | 1. **Limited tracking capacity** | - *We initially requested the serial numbers but then decided not to record them - it is very time consuming and uncertain about the clinical relevance. It was a lot of work for a small practice. So we opted instead to record the cuff sizes. [PM]* - *practices do not have the manpower or infrastructure to keep an accurate log of [BPMs] [GP]* - *I suspect the biggest problem is going to be that we've got is it 16,000 monitors across NCL and a lot of those are going to disappear because of the way it's managed. (…) I'm worried about the investment that's gone into this. And where that will be in, you know, a year or two years' time. (…) So I'm not saying that they'll be lost in terms of that they'll disappear, but they'll be with patients and practices will not know which patients have got them anymore. {GP]* |
|  | 1. **Lack of awareness & unification of IT tools** | - *I was not aware of apps and other IT support so I created a spreadsheet with each patients' average BP. If high, I give them a phone call (…)_Readings are both on EMIS and spreadsheet but I didn't know how to have the 2 communicating [PM]* - *We had to create templates at ICS or PCN level (for EMIS, onboarding, etc) [PM]* - *without [a template created by one of the respondents], it would just have been an absolute nightmare because there was nothing in place at all when we started doing this and all the PCNS we have onboarded have got it [PM]* - *one of the issues we currently have is perhaps too much choice in too disparate ways. So by that I mean if we're onboarding patients and we're giving them a choice of ways to give us blood pressure readings, each practices having to kind of work out, how do they make that available for their staff. So you know each individual doctor might have a link to the printable blood pressure, you know, diary to give to a patient, but some of them don't have that and they don't know where to find it. [PM]* |
|  | 1. **Limitations of existing tools** | - *the problem with AccurX is that is sounds as if it is text based but it actually requires a smartphone (…) And we find that quite a lot of our AccurX requests fail either because the patient doesn't want to bother taking their BP for 4 days or because they just can't manage the tech.* - *the remote monitoring requires the patient to at least have a mobile phone. So we have some older patients (…) or actually patients who maybe are from the slightly more deprived backgrounds who have mobile phones but don't have Internet access on their mobile phone. [PM]* - *Links expire at the end of the 4 days so if patients don't use it right away, it is lost* - *[the Hypertension Plus Omron app) is very similar but unlike BP@home, the app sends back continual info to patients, creating a sort of interactive dialogue. The onboarding process is not necessarily easier but once it is completed, it appears stickier. [PM]* |
| **Recruitment / engagement of PCNs & practices** | 1. **Differential capacities** | - *PCNs are very variable beasts: say in some areas a PCN is a group of practices working very, very closely together and I think. Then you can get a whole program out across the PCN, but actually in a lot of places a PCNS are a bunch of practices forced to work together in order to get their funding streams in, but they're not actually really working together [PM]* - *skill sets vary from practice to practice [PM]* - *Capacity within primary care is the main challenge [PM]* - *Getting dedicated time to do the work has definitely a barrier in many of the practices. [PM]* |
|  | 1. **Substantial additional workload** | - *an extra piece of work (…) it's too complicated, we haven't got the time to do this [PM5]* - *Broadly speaking, the initiative was highly welcomed. However, due to pressure from implementing vaccine rollout for Covid-19, resources were redeployed [PM]* - *because it seemed like to practices "oh no, this is a lot of to do" because they've got to store the blood pressure machines, they got to keep a track of who's using them and there is also some training element for staff and an influx of information. Whenever you send that request you get all these responses coming back [PM]* - *It requires a lot of admin and "old school chasing". It is a full-time job to then chase the readings. It is an "ongoing chase of results" [GP]* |
|  | 1. **Lack of financial incentives** | - *The only financial incentive for this is actually getting the monitors. There's no financial gain for a PCN, and that's why it's such a a huge sort of issue, because PCN's are looking at well as this project going to make us money [PM]* - *There's no direct funding. [PM]* |
| **Recruitment / engagement of patients** | 1. **Limited patients' interest** | - *In some in some areas where they've been very focused about getting blood pressure machines out to patients (…) I'd be interested to see whether that's still as successful as it was at the beginning. Because my experience is that we can get monitors out to patients, and we can get them to give us some readings some of the time and not all of the time. And then it's another big piece of work to keep them engaged in that (…) move towards BP at home being a thing that the patients are prompting [PM]* - *It went on for too long and patients lost interest. It might have helped to have a timeline. If we don't dress it up as a trial, patients might take it more seriously [PM citing feedback received from other care coordinators in the local PCN]* - *Patients didn't need much help with using the BPM, they were quite comfortable using them. But many did not follow the request to send 4 readings a day, even after being sent a reminder and being provided a diary in the letter. [PM]* - *Some felt there might be more pressing things than their BP: mostly getting a jab [PM]* |
|  | 1. **Patient least engaged are those needing most care** | - *Patients who self-manage and know their BP is ok sometimes stop engaging with us and don't submit readings [PM]* - *Higher BP patients were the ones not sending their readings, maybe they didn't like seeing the results. The ones sending most readings were the ones needing least care. [PM]* |
|  | 1. **Equity concerns** | - *there are PCN that have had lots and lots of monitors and their patients are benefiting, and you're having these populations who have high levels of deprivation, who again have missed out on these BP monitors. And so again, that gap between, like you're keeping the well, really well and you're still doing a disservice to the patients who are deprived [PM]* |
