# Supplementary material for: Investigating barriers & facilitators for the successful implementation of the BP@home initiative in London: Primary care perspectives
Source: PLoS One. 2024 Feb 29;19(2):e0298898. doi: 10.1371/journal.pone.0298898 (PMC10903909; doi:10.1371/journal.pone.0298898)
Supplement: S4 Table — (DOCX) [file pone.0298898.s006.docx]

**S4 Table: Thematic analysis of strategies & enablers identified by respondents (with supporting quotes)**

| **Categories** | **Themes** | **Key Supporting Quotes** |
| --- | --- | --- |
| **(1)**  **Project Management** | **Good comm with NHSE & within ICSs** | - *Communication was very good throughout the initiative. [PM]* - *We had regular meetings with NHSE. Report had to be at ICS level. So we had to come together to make decisions. Ongoing discussion around what process and systems look like, avoid duplicating things.[PM]* - *There have been a couple of meetings of our ICS Community of Practice to date where learning about implementation has been shared by the early adopter sites [PM]* |
|  | **Centralisation at practice & PCN levels** | - *The most important thing that will drive the success of this or not is to have a named person for every practice who is trained and most importantly funded to do this because it takes time and the practices that are successful with BP at home are the ones who have a person who has allocated time to be the responsible. Because what happens then is that practice, you know, if a GP has a patient who they think would be suitable for BP at home. They just messaged that person and say can you on board them? It's a very quick process because GP's don't have time to do anything more than that. So if you have someone who they can just pass ownership to, who can then on board the patient, that's what works the best. [GP]* |
|  | **Task-sharing & coordination focused on LTCs** | - *that PCN is quite well organized. So they have a long term condition clinical lead who is a GP and then that GP has a team that they works with. So there's a lead pharmacist for long term conditions alongside a computer IT guru. So he's just for long term conditions. So that was brilliant. So that was really helpful. We had a reception representative. So she was feeding back all the queries and concerns from a front facing reception point of view. How this is gonna work for practices? What training do we need for the actual reception teams? How's it gonna work specifically for those frontline staff? And then we also had a PR person on that team as well. What information do we need to put on the website? What information do we need to send out to patients? So they're very well organized [PM]* - *So there is someone being appointed as the NCL LTC clinical lead and their job is basically going to be to go to practices and try and sort of establish what the problems are and what support they need, what resources they need, what's lacking in certain practices and PCN [PM]* |
|  | **Incorporation into daily practice & reverse-thinking** | - *I would say that we make this part of our everyday consultation and this hasn't seen as an added project. This is seen as something that we incorporate into our day-to-day long term condition reviews [PM]* - *It would help to make it part as business as usual (…) and then maybe not be tied so tightly to the UCLP searches because everyone has their own kind of popup list that they work on [CP]* - *it is here to stay. It is a priority as part of pushing proactive care. I do think it can become more and more sustainable, but we just need to keep tweaking and refining how we integrate it into our everyday approach to patients with long term conditions and also how we support patients in their engagement, especially for groups who have been hard to engage [PM]* - *for this to be successful actually practices need to start at the end of the process, not at the beginning. And what I mean by that is every practice that wants to engage with supporting BP at home needs to solve the problem of how will their data come in? Who will manage it and what will happen when the patients blood pressures are not well controlled and that's actually the first step of the process [PM]* |
| **(2)**  **Logistics** | **BPMs on prescription / on loan** | - *In one ICS, we chose a plan of recycling machines 3 to 4 times due to concerns about equity (avoid 1^st^ arrived, 1^st^ served logic): just taking them out and walking away felt irresponsible. Tier 3 for a week. Tier 1 & 2: 8/10 weeks & up to 6 months [PM]* - *Many of us feel that the process of loaning blood pressure machines may be better moved to prescriptions - a routine part of managing hypertension and some money coming back for those who can afford from prescription charges. May improve the balance of who gets devices also [PM]* - *There's a whole process that is taking us a lot of time, taking the machines out, etc. which is non-sense. There is always a risk for all the patients we lend a BPM to, of them not using them but it would still be cost-efficient: 10£ each when bought in bulk might save a lot on medication. It is a no brainer. Patients might not use them at first, but they will have them for the future and might use them later on [N]* |
|  | **Development of practice-specific processes** | - *Systems for managing results have to be developed for each practice as all practices work differently and it has to be decided who will respond to results etc. Some of these resources we have shared with practices existed on the NHS futures website and have been adapted for use locally [PM]* - *a lot of processes to be thought about and developed. And it still does for any practice because it happens at practice level: each practice needs to have systems for managing things they didn’t use to manage, which is patients sending up results from home, and also onboarding them, giving equipment, etc. [PM]* - *There was a list that UCLP had initiated to say well, look, these are the sorts of metrics you might want to track to give you an idea about how well this is working. So how many machines are out on loan? How many requests are you sending out versus how many blood pressure submissions are coming back in so that you get a rate of of success response I suppose? And then how many new consultations are happening as a result of those new blood pressure submissions? So we were able to work out some set UCLP had given us initially some searches and we created some local searches as well with the IT guy and then he just kind of runs those searches on a fortnightly basis and we can keep track of how that's going. And so that was good. [PM]* |
|  | **Simplified templates & IT system** | - *we've brought together quite a lot of resources, but actually it would be quite nice to have them much more streamlined and easily available. Maybe adapting the template and simplifying* ***it****, but with links to the resources that people need so you know a simple letter that explains to the patient how to do this (…) It is quite a lot of different bits and pieces and maybe somebody with a really good head for simplifying things could bring it together. [PM]* - *If someone can come up with a system and very simple way of monitoring where our blood pressure machines go, so then we can keep you know a recalling those patients that would be great. [CP]* |
|  | **Engage with pharmacists** | - *the pharmacist were very good at thinking about the* *logistics of how the review is gonna take place in the consultation. How will we know that the patient has had the right size cuff? How will we know that the patient is taking the blood pressure readings in the way that we want them to? So making sure that we had good information going out to patients in the initial messaging that went out and the batch messages, but also that we had a check process, so that all the clinical pharmacists were in the PCN knew to double check with the patient once they so a patients readings would come in, they would be allocated in appointment to phone that patient to discuss the results. And there was a checklist of let's just double check. Are they using the right size cuff? How are they doing it? And I suppose validate the results a little bit more. [PM]* - *The pharmacists are amazing. So if you can get your pharmacist on board, they're brilliant at motivating patients as well as making sure that data is being recorded in the right way so that it's useful for them, for your IT people to pick up things on their searches and things like that. So I think that's really important. [PM]* |
| **(3)**  **Engagement of PCNs & practices** | **Adequate / additional funding** | - *I set up an incentive scheme as we recognized that money would be helpful. 130K so about 15K per borough for the 8 boroughs, about 2 or 3K per PCN, so very little per practice but it smoothed an awful lot of feathers and was very helpful. It also gave an opportunity to provide a plan: they had to put up a PCN team, (…) and confirm that 10 unique patients had submitted readings remotely to receive the incentive [PM]* - *work like this is very difficult to implement without funding it (…) a lot of the work that's expected to be done is sort of out of an already stretched system that doesn't have actually capacity. And so projects like this just need to be adequately resourced. [GP]* |
|  | **Clinical targets as incentives** | - *Right from the beginning, the incentives were the important part because that was the way to try to get practices on board because it seemed like to practices. Ohh no, this is a lot of to do because they've got to store the blood pressure machines they got to keep a track of who's using them and there is also some training element for staff and an influx of information. Whenever you send that request you get all this responses coming back so incentive wise, it was about tying it to targets that they already have. [PM]* - *For example, QoF definitely, so we could demonstrate how this is gonna help them to reach their quaff targets. Great. That's a big tick. The other one is there's a there's a local locally commissioned long term conditions service that Enfield Borough already had had in place. So they were like in year two of their cycle. And they had some very tight hypertension goals on there. That practices had been struggling to achieve. So where we could say, OK, look, you're trying to achieve this for your blood pressure. Here's where you are so far. What if we can get you here by trying this method and then OK, now they're happy to try. [PM]* |
|  | **Needs assessment & training at ICS level** | - *skill sets vary from practice to practice. So we did a little bit of a needs assessment with the PCN and said, look, what do you think you're gonna need within your practices? There were some places where the pharmacists were quite junior, so we did some specific training with the pharmacist, had a few sessions with them before the patient launch so that they felt competent to go ahead. They knew what to do. We did a lot of role-playing that sort of thing. The receptionist representative did some training with reception team, so we standardize materials that were sent out to all reception teams across the practices. And then she did some sort of follow up training with them so that they felt comfortable how to approach the patient. [PM]* |
| **(4)**  **Engagement of patients** | **NHSE onboarding material & integration of UCLP into EHR** | - *Material was* *fantastic, extremely clear, a lot of support with IT, how to explain to patients that it would benefit them, and how. Integration with EMIS worked very well. No unanswered questions, we didn't feel like we were in the dark. I created my own scripts based on those documents and my own experience [N]* - *I have found the BHF materials useful.... and the new updated UCLP <2min video is helpful too [GP]* |
|  | **Pragmatic & opportunistic approaches to onboarding** | - *We got around 800 patients. As pilot we tried different things. The GP is in charge of discussing it with the patient or request me to call or set up an appointment with the patients. It was mainly opportunistic. Following UCLP [we came up with] a lot of patients and 30 minutes chat were not feasibile. We shortened to 15 minutes. Small practice so quite intimate [CP]* - *If we have only 20 machines, there is no point sending 200 texts. Only send 20 texts. Saying we identified you to benefit from this program. If patient sends back "yes", call them back. (…) Pragmatically, we simply called back those who responded yes [PM]* - *"What we did was slightly different. We looked at the priority list & then matched them with high-risk patients who have pre-existing diabetes so the 3Ts as we call them. Then we sent en masse text messages to all the patients in the cohort to identify those who have a BPM at home. (…) What we found is that we had a good proportion of patients who already had a BPM. So we then started to engage in terms of creating a management plan that is unique to* *our practice in that it included information about blood pressure, about the medication that they were taking, along with instructions about how to monitor BP at home". [CP]* |
|  | **Person-centred care** | - *Patients have to understand why we're doing it and that is not just a thing the practices is asking for the benefit of the practice. They need to see that it's personally helpful for me to understand my blood pressure and to have this engagement and interaction with the clinician. [PM]* - *When they buy into the fact that you're not just doing this as a tick box, you actually care about helping them achieve something they're more likely to be willing to to engage.[PM]* - *[BP] is one of those things you're not aware of necessarily, and so helping the patient to realize, hey, there's something you can do to help protect your health. We're here to support you with that. And we will give you whatever support you need, but you can do this to help with your health. And we can guide you with that process. I think that's really beneficial because it has positive knock-on effects to other health areas because if a patient is quite used to monitoring their own blood pressure, they're going to be more likely to look out for foot ulcers if they've got diabetes or to look out for ABC [PM]* - *for those patients who think oh I'm great, I think it's good. What we did was built in a feedback loop with loop with the Florey* that if their blood pressure was to target that we said OK, you don't need to do this for another 12 months and we automated it. So it would contact the patient in 12-month time because we don't want patient fatigue. So as likely as you say if they're being asked to do something all the time and they keep getting told, it's fine, it's fine. It's fine. At some stage they may well just give up. Plus, it's not that useful clinically if they are well controlled. We check again in a year [PM]* |
|  | **Diversity of communication channels to fit each patient** | - *the remote monitoring requires the patient to at least have a mobile phone. So we have some older patients (…) or actually patients who maybe are from the slightly more deprived backgrounds who have mobile phones but don't have Internet access on their mobile phone. So that was OK because we sent the initial messages just as an SMS text message with the information and they could reply to the message if they wanted to bring their results in on paper. So we did have a cohort of patients who don't want to use Floreys* for whatever reason. They either got confused using a Florey* or they didn't have Internet access or their mobile phone was a more basic mobile phone without Internet access. So depending on the area, but it was usually not more than 10% of patients were still bringing paper copies in, but we still use the same protocol, the same SOP, to monitor them and so on.[PM]* - *we use 4 different methods of sending in their BP readings: they can hand them at the reception by hand, they can email them to the practice email address, they can email the Excel spreadsheets that averages them for us [GP]* |

* Florey is a feature in Accurx designed for collecting structured data and screen/monitor patients remotely. See this link for more details <https://support.accurx.com/en/articles/3542649-florey-what-is-florey>
