# Supplementary material for: Investigating barriers & facilitators for the successful implementation of the BP@home initiative in London: Primary care perspectives
Source: PLoS One. 2024 Feb 29;19(2):e0298898. doi: 10.1371/journal.pone.0298898 (PMC10903909; doi:10.1371/journal.pone.0298898)
Supplement: Attachment — Submitted filename: Response to Reviewers.docx [file pone.0298898.s007.docx]

**Response to Reviewers - PONE-D-23-22751**

**Investigating barriers & facilitators for the successful implementation of the BP@home initiative in London: primary care perspectives**

Dear editor & reviewers,

Thank you very much for your thorough & valuable review of this paper. Please see below our responses to each of your comments.

**Editor**

|  | **Comments** | **Responses** |
| --- | --- | --- |
| **1** | Please ensure that your manuscript meets PLOS ONE's style requirements, including those for file naming. | Thank you. We have revised the manuscript following PLOS ONE’s style requirements, including those for file naming. We also removed funding or competing interests information at the end of the manuscript and only kept the Acknowledgments section. |
| **2** | We note that you have indicated that data from this study are available upon request. PLOS only allows data to be available upon request if there are legal or ethical restrictions on sharing data publicly. For more information on unacceptable data access restrictions, please see <http://journals.plos.org/plosone/s/data-availability#loc-unacceptable-data-access-restrictions>.  In your revised cover letter, please address the following prompts:  a) If there are ethical or legal restrictions on sharing a de-identified data set, please explain them in detail (e.g., data contain potentially sensitive information, data are owned by a third-party organization, etc.) and who has imposed them (e.g., an ethics committee). Please also provide contact information for a data access committee, ethics committee, or other institutional body to which data requests may be sent.  b) If there are no restrictions, please upload the minimal anonymized data set necessary to replicate your study findings as either Supporting Information files or to a stable, public repository and provide us with the relevant URLs, DOIs, or accession numbers. For a list of acceptable repositories, please see <http://journals.plos.org/plosone/s/data-availability#loc-recommended-repositories>.  We will update your Data Availability statement on your behalf to reflect the information you provide. | Thank you for raising this. After checking the following guidance provided on PLOS website: “For studies analyzing data collected as part of qualitative research, authors should make excerpts of the transcripts relevant to the study available in an appropriate data repository, within the paper, or upon request if they cannot be shared publicly”, we believe that Supplementary files S5 and S6 do contain the minimal anonymized data set necessary to replicate our study findings. In fact, these include all supporting quotes for each identified theme.  We have revised our cover letter to reflect this by adding the following sentence: “The minimal anonymized data set necessary to replicate our study findings have been provided as Supporting Information files.” |
| **3** | Your ethics statement should only appear in the Methods section of your manuscript. If your ethics statement is written in any section besides the Methods, please delete it from any other section. | Thank you. We have removed the “Ethics” section at the end of the paper (after the Conclusion). |
| **4** | We notice that your supplementary figures and tables are included in the manuscript file. Please remove them and upload them with the file type 'Supporting Information'. Please ensure that each Supporting Information file has a legend listed in the manuscript after the references list. | Thank you. We have removed our supplementary figures and tables from the manuscript file and saved them as individual Supporting information files. |

**Reviewer 1**

| **Section** | **Comments** | **Responses** |
| --- | --- | --- |
| **Abstract** | The abstract needs to be more concise. | Thank you. We have revised the abstract to make it more concise. |
| **Strengths & limitations** | Please define separately. | Thank you for this suggestion. We have now presented the strengths and limitations in two separate sub-sections of the discussion. |
| **Conclusion** | Recommendations need to be addressed separately. | Thank you for this suggestion. Authors’ recommendations are addressed separately, along with the implementation implications. |
| **General** | The article should be grammatically sound. | We have further revised the paper to ensure it is grammatically sound. |

**Reviewer 2**

| **Section** | **Comments** | **Responses** |
| --- | --- | --- |
| **Methods** | The description of the data collection methods is not fully clear. a. Were 5 focus groups and 1 one-on-one semi-structured interview conducted? If focus groups were used, authors should describe key characteristics of the focus groups, per generally accepted guidelines. For example, how many people attended each focus group; where focus group attendees homogenous in terms of occupation to facilitate frank discussions? Who facilitated the focus group discussions? I suggest the term “interviews” be used only for interviews and not for focus groups, as focus groups constitute a method different from interviews. | Thank you for your comment. We have provided additional details regarding the focus groups, including who lead them and who were the participants, both in the text (“All focus groups were facilitated by AEO”) and by the addition of “**Table 1**: Composition of Focus Groups”.  We also revised the wording across the manuscript to use “focus groups and interviews” rather than just “interviews” where appropriate. |
|  | b. Quotes in the result section and S5 and S6 Appendices should specify the respondents’ role, to the extent doing so does not compromise anonymity. | Thank you for noting this. All quotes in the manuscript and supplementary files specify the respondent’s role. We had initially thought of numbering them (e.g. GP 1, PM 4, etc) but thought this might compromise anonymity. Therefore, only the role has been specified (as suggested), considering that this is sufficient to “situate” each quote’s perspective without compromising anonymity. |
|  | c. Please cite your methods. | Thank you. We have specified the references supporting our methodological choices, including the data collection tools, sampling methods, and the analytical framework supporting data analysis. |
|  | d. Once methods are clarified, strengths and limitations should reflect these methods. | We have added some limitations and strengths related to the methodological choices we made. |
| **Results and discussion** | I think the results and discussion sections would be enhanced by a conceptual framework, e.g., an implementation framework. For example, do logics/project management factors map to an “early” stage of implementation whereas patient onboarding stage factors to a “later” stage?  Furthermore, it is not clear what is meant by “system level” per the paragraph about the aim of the study? Does “system level” denote NHS level? Or do the authors mean different system levels, e.g., ICS level, PCN level, GP level? Which barrier/facilitator is “system” level? Which barrier/facilitator is at the ICS level versus GP level, if such a distinction is reasonable? The authors should be clear about what is meant by “system level”, and a conceptual framework may help with this clarification. | Thank you for your suggestion. We used CFIR to contextualise our findings, by mapping them to key CFIR constructs, and to help guide the development of congruent recommendation’s to streamline the delivery of BP@home initiative |
|  | I think the authors should also describe implementation implications based on the combined findings of this study and recent studies (references 15-22). | Thank you. We added a section in the Discussion entitled “**Implementation implications & authors’ recommendations”** where we describe the implications of our findings, combined with recent studies, on the implementation of BP@H. |
| **Minor issue** | These are some words that are difficult for an American reader to understand, e.g., “crib sheet” (Interview guide?), and from S6 Appendix, “Flory” and “flurry”. | Thank you for your comment. We have replaced “crib sheet” by “interview guide” across the manuscript and supplementary information. We also added an asterisk and explanation of what a Florey is: * Florey is a feature in Accurx designed for collecting structured data and screen/monitor patients remotely. See this link for more details <https://support.accurx.com/en/articles/3542649-florey-what-is-florey> |

**Reviewer 3**

| **Section** | **Comments** | **Responses** |
| --- | --- | --- |
| **General** | First and foremost, I commend the authors for this insightful study that delves into the barriers and facilitators for the successful implementation of the BP@home initiative. The manuscript is coherently written and follows an organized format. Furthermore, the adherence to COREQ guidelines is a commendable aspect of your methodology. | Thank you for your supportive statement which is much appreciated. |
| **Methods** | It's mentioned that a total of 20 individuals took part in this study. Could you elaborate on how this sample size was determined to be sufficient? The manuscript states that the sample size of 20 was felt adequate to achieve saturation. However, it is not explicitly clarified whether data saturation was indeed reached. | Thank you for this comment.  We have added a sentence regarding this in the Data Collection section: “Of those who responded and agreed to take part, two eventually could not attend any of the focus groups scheduled due to time constraints. However no additional interviews or focus groups were organized as theme saturation had already been achieved.”  We also rephrased our sentence in the Strengths section as follows: “In addition, we feel that saturation of themes was achieved, as recommended in qualitative studies [[47](https://bmcpublichealth.biomedcentral.com/articles/10.1186/s12889-022-14426-9#ref-CR47)], since no new themes appeared in the last focus groups.” |
|  | Can you specify how many individuals were approached for the interview? It would be beneficial to know if everyone agreed to participate or if some declined or opted out during the process. | Thank you for this comment. We have clarified this point in the Data Collection section. |
|  | Regarding the relationship with participants: It would enhance the rigor of your study if you can clarify any pre-existing relationships between the data collectors and the participants to ensure transparency about potential biases. Were the interviewers known to the participants, or were they neutral entities? | Thank you for noting this. We have specified that “3 participants were previously known to study authors by virtue of participating in a local clinical research group monthly online meetings.” |
| **Findings** | Under the "Barriers" section, the discourse related to the theme of "project management" suggests that the study primarily focused on evaluating the BP@home program. There seems to be a slight divergence from the initial goal, which was understanding impediments to its implementation. For instance: How do the shifting direction and the reduced timeframe for the project's roll-out impact the program?  Were there any repercussions due to delays in the project's implementation? It would enrich the findings if you could provide insights into how such challenges were managed or addressed. | Thank you for highlighting this. We added a section “implementation implications & authors’ recommendations” which aims to clarify these aspects. We have also contextualised our findings using the CFIR frameworks, mapping them to key CFIR constructs. |
